# Antioxidative effects of cerium dioxide nanoparticles ameliorate age-related male infertility: optimistic results in rats and the review of clinical clues for integrative concept of men health and fertility

**DOI:** 10.1186/s13167-015-0034-2

**Published:** 2015-06-10

**Authors:** Nazarii M. Kobyliak, Tetyana M. Falalyeyeva, Olena G. Kuryk, Tetyana V. Beregova, Petro M. Bodnar, Nadiya M. Zholobak, Oleksandr B. Shcherbakov, Rostyslav V. Bubnov, Mykola Ya Spivak

**Affiliations:** Bogomolets National Medical University, T. Shevchenko boulevard, 13, Kyiv, 01601 Ukraine; Taras Shevchenko National University of Kyiv, Volodymyrska Str., 64/13, Kyiv, 01601 Ukraine; State Scientific Enterprise “Scientific Practical Center for Prophylactic and Clinical Medicine” State Management of Affairs Department, Kyiv, Ukraine, Verhnya str., 5, Kyiv, 01014 Ukraine; Zabolotny Institute of Microbiology and Virology, National Academy of Sciences of Ukraine, Zabolotny Str., 154, Kyiv, 03680 Ukraine; LCL “DIAPROF”, Svitlycky Str., 35, Kyiv, 04123 Ukraine; Clinical Hospital ‘Pheophania’ of State Management of Affairs Department, Zabolotny Str., 21, Kyiv, 03680 Ukraine

**Keywords:** Predictive, preventive, and personalized medicine, Nanocrystalline cerium dioxide, Age-related male infertility, Oxidative–antioxidative balance, Infertility biomarkers, Seminal plasma, Ultrasound, Translation, Men health

## Abstract

**Background:**

Male infertility has largely idiopathic, multifactorial origin. Oxidative stress is a major factor that affects spermatogenesis, in particular in aging. Cerium dioxide nanoparticles (CNPs) due to their antioxidative properties are promising to impact on the development of male infertility. *The aims* of this study were to investigate the effects of CNPs on fertility parameters in 24-month male rats and to overview relevant literature in the field of personalized treatments, predictive diagnosis, and preventive measures for male health and fertility.

**Methods:**

We included 30 24-month-old male rats. After a week of adaptation to the standard diet, the rats were randomly divided into three groups with ten rats in each. Group 1 (controls) received only a standard diet. The rats of group 2 and 3 in adjunct to the standard diet during 10 days received intragastrically 10 % sodium citrate and citrate-coated CNPs in dose 1 mg/kg, respectively. We assessed sex hormones, epididymal sperm parameters and spermatogenesis, ultrasound, and morphological data of rat reproductive organs.

**Results:**

After a 10-day administration of CNPs, we revealed significant decrease of lipid peroxidation product levels in serum and increase of catalase and SOD activity, associated with increase of sperm count (*p* < 0.001) and improvement in quantitative sperm parameters (motility, viability, and percentage of spermatozoa). We found no significant changes between sperm quantitative parameters in citrate-treated rats and controls and observed age-related decrease of activated Leydig cell number and focal atrophy of the seminiferous tubules. In CNP group, we observed regeneration of seminiferous tubules, increase number and activation of Leydig cells, and 2.5-fold significant increase of serum testosterone. Ultrasound data showed the slight increase of linear measurement and volume of rat testes in CNP group. Review highlights the benefits for predictive diagnosis, preventive measures, and personalized approaches to manage male infertility in the general concept of male health also related to aging.

**Conclusion:**

Citrate-coated 2–5-nm CNPs lead to increase in sex hormones levels, sperm count, and quality, as well as the activation of spermatogenesis in 24-month-old male rats. Nanoceria demonstrated the perspectives to be an effective infertility treatment via reduction of oxidative stress in male reproductive organs, in particular in aging.

## Overview

### Predictive, preventive, and personalized medicine for male infertility. Aspects of oxidative stress—challenge for nanomedicine

The tasks of *predictive*, *preventive*, *and personalized medicine* (PPPM) is to develop well-balanced family life through all life spans in aging society and promote sustainable reproduction health and healthy new generation. The issue of the health of elderly in aging society and fertility of males is the crucially important task for medicine of the future which is focused in the separate paragraphs of granting research programs.

**Fig. 1:**
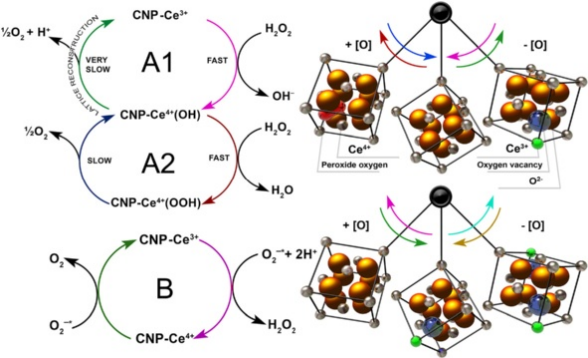
Schematic model of catalase-like (*A1*, *A2*) and superoxide dismutase-like (*B*) activities of CNP (left) and corresponding changes in ceria lattice [2] (right)

Cerium dioxide (CeO_2_) nanoparticles (CNPs) have shown promise as a therapeutic application due to their antioxidant auto-regenerative ability and low toxicity [1, 2]. The electronic structure of CNPs at the nanoscale leads to their antioxidant activity. Both large surface-area-to-volume ratio with the reduction in particle size [3, 4] and ability to reversibly switch between Ce^3+^ and Ce^4+^ present on the surface [5, 6] result in the formation of oxygen defects in the crystal lattice that act as “reactive sites” or “hot spots” for free radical scavenging [7]. A schematic model explaining CNP surface regeneration and its activity is shown in Fig. 1. The combination of auto-catalytic and regenerative properties makes CNPs desirable therapeutic agents [8, 9].

The recent studies confirmed that CNPs exhibit superoxide dismutase (SOD) [10, 11] and catalase mimetic activity [12] and also have the ability to scavenge hydroxyl (·OH) [13] or nitric oxide (·NO) radicals [14]. Several reports demonstrated that CNPs with size less than 10 nm, regardless of the dose, did not induce overt toxicity or immune response and are well tolerated by the experimental animals [15, 16].

Reactive oxygen species (ROS) can exhibit a dual effect on the male reproductive system. In a healthy organism, small amounts of ROS are produced by spermatozoa and they play an important role as key signal molecules which are involved in capacitation and the acrosome reaction [17, 18]. The balance between their production and inactivation by various scavengers is strongly controlled due to physiological conditions [19]. Violation of this balance occurs with aging and under various reproductive disorders which lead to enhanced ROS formation or impaired mechanisms of antioxidant production results in oxidative stress [20]. In both human and animal studies, the role of oxidative stress within sperm was emphasized, resulting in DNA damage [21, 22], reduced sperm parameters [23, 24] and gene expression [25, 26], and defective membrane integrity [19].

In our preliminary study, we revealed that CNPs (2–5 nm) in dose 1 mg/kg had a stimulating effect on spermatogenesis in 18-month male rats. CNPs at a dose 100 mg/kg had no significant effect on the assessed indices of the morpho-functional condition of reproductive system [27].

Men health in the point of view of reproduction, personal health, and life quality is still not sufficiently studied and implemented in predictive assessment and widespread screening programs.

Therefore, *the aims* of this study are to investigate the effect of CNPs on qualitative and quantitative sperm parameters, spermatogenesis, sex hormone levels, and pro/antioxidant condition in 24-month male rats, to overview relevant literature in the field of personalized treatments, predictive diagnosis, and preventive measures for male fertility, and to suggest the translating and implementation of obtained data in the concept of predictive, preventive, and personalized medicine.

## Methods

### Synthesis of citrate-coated CNPs

Colloid 0.1 M solutions of CNPs stabilized by 0.1 М ammonium citrate were synthesized as follows [28]: 3.73 g of cerium (III) chloride heptahydrate and 2.0 g of citric acid were dissolved in 20 mL of distilled water. Under continuous stirring, this solution was added rapidly to an aqueous ammonia solution prepared by mixing 10 g of concentrated ammonia solution (Sigma, USA) and 100 mL of distilled water. The resulting mixture was stirred for 5 h, with further boiling aiming at producing 100 mL of 0.1 M ceria sol. Finally, the sol was purified by precipitation-redispersion with isopropyl alcohol and boiled.

Optical absorption spectra were recorded on an OceanOptics QE 65000 spectrometer using a one beam scheme. The radiation sources were a DH 2000 deuterium–halogen lamp and an HPX 2000 xenon lamp. According to UV-vis spectroscopy data, the bandgap for CNPs is 3.5 eV. Upon long-term storage, the bandgap of CNPs stayed unchanged, thus indicating that the particle size in ceria sols is constant.

**Fig. 2:**
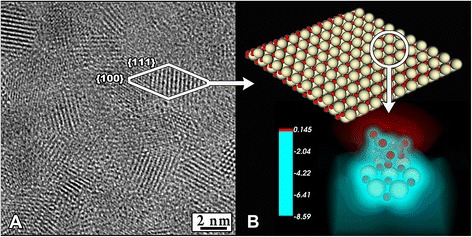
High-resolution electron micrograph (HRTEM) of ceria nanoparticles (4–5 nm) stabilized by citrate (**a**). The model of lattice and 3D spatial distribution of the effective potential of the citrate anion adsorbed on the cluster Ce_7_O_12_ (**b**) [1]

The size of CNPs was determined by transmission electron microscopy (TEM) on a Leo 912 AB Omega electron microscope at 100 kV accelerating voltage. According to TEM micrographs and electron diffraction data (Fig. 2), the solution consists of weakly aggregated CNPs of nearly isotropic shape 2–5 nm in size.

The hydrodynamic diameter by the dynamic light scattering (DLS) method and zeta potential of citrate-coated CNPs was measured on a Malvern Zetasizer Nano ZS analyzer. Before the measurements, the sol was diluted with distilled water. DLS data indicate that mean hydrodynamic diameter of CNPs is 4.9 nm. This is consistent with the TEM data and is evident that the presence of a monomolecular (or sub monomolecular) layer of citrate ions has no noticeable effect on the particle size. Zeta potential of CNPs is negative (−20 mV).

Powder X-ray diffraction (XRD) analysis of ceria nanopowders prepared by sol centrifuging was carried out on a Rigaku D/MAX 2500 diffractometer (CuKα radiation, instrumental broadening 0.10 ± 0.01°2*θ*). The goniometer rotation speed was 2°2*θ*/min. Crystallite size (*D*) of nanocrystalline ceria was calculated using *Scherrer* formula where the coefficient of anisotropy was set to 1. Line profiles for (111) and (200) reflections were fitted to pseudo-Voigt functions. XRD has shown that the particles separated from the sol by centrifuging are single-phased and correspond to cubic CeO_2_. Analysis of diffraction peaks broadening indicates that the synthesis method proposed allowed us to obtain ceria samples with crystallite size about 3 nm.

### Study design

After 1 week of adaptation to a standard diet, 24-month rats were randomly divided to one of the three groups with ten rats in each group. Group 1 (controls) received only a standard diet. Rats of group 2 and 3 in adjunct to standard diet were administrated intragastrically during 10 days with 10 % sodium citrate or citrate-coated CNPs in dose 1 mg/kg, respectively.

After 35 days from starting the experiment (about one duration of spermatogenesis), all rats from three experimental groups (*n* = 30) were killed by cervical dislocation under urethane anesthesia. Immediately after dissection, testis, epididymis, ventral prostate, and seminal vesicle (without the coagulating gland) were removed and weighted (absolute and relative to body weight). Relative organ weight was expressed as organ weight (mg)/body weight (g).

### Sample collection and blood biochemistry analysis

Blood was drawn from the apex of the cardiac ventricle, and few blood drops were collected into a microcentrifuge tube containing a mixture of NaF and EDTA in a 2:1 (*w*/*w*) ratio. The blood sample was collected into a sterile tube and centrifuged at 3,500 rpm (2260 g) for 15 min. After centrifugation, serum supernatant for further analysis was aliquoted into microcentrifuge tubes and stored at −80 °C. ELISA was used for determination of serum testosterone and luteinizing hormone (LH) level by commercial kits «BioVendor» (Czech Republic).

### Sperm parameter assessment

Sperms acquired from the tail of the left epididymis were released to 2 ml of saline (0.9 % NaCl). After 15 min of incubation, one drop of sperm suspension were removed to Goryaev counting chamber, covered by a 22 × 22-mm coverslip to estimate sperm count, viability, motility, and morphology according to WHO protocols [29]. Under a light microscope, Olympus BX51 (Olympus Optical Co. Ltd., Tokyo, Japan) at ×400 magnification, the total sperm counts were calculated and the value was expressed as ×10^6^/mL.

At least 200 sperms for each specimen in the minimum of five microscopic fields were assessed to evaluate sperm motility. Sperm motility was expressed in percentages and categorized as follows:

- Grade A—registration of rapid motility (movement in straight lines with a speed of not less than 25 μm/s or cover the distance equal to its length for 2 s).
- Grade B—slow motility (movement in straight lines with a speed less than 25 μm/s). Grade A + B according to the WHO protocol (2010) classified as “sperm with progressive motility”.
- Grade C—nonprogressive motility (spermatozoa do not move forward despite the movement of their tails).
- Grade D—immotile sperm (fail to move at all).

For evaluation of sperm viability, a drop of sperm suspension was mixed with an equal volume of 0.05 % eosin Y. The mixture was incubated at room temperature for 2 min and analyzed under a light microscope, at ×400 magnification. Dead sperm cells were stained red while live sperm cells were unstained. A total of 100 spermatozoa per sample were counted and part of viable sperm were expressed as the percentages.

The sperm was stained with hematoxylin for morphological anomaly assessment. A total of 100 spermatozoa from different fields were classified in next general categories: such as normal, abnormalities of the sperm head (abnormal shape, size, double or triangular head, absence or reduced acrosome), or abnormalities of the flagellum (bending, lack or plural number of flagella, abnormal size). The sum of abnormal spermatozoa was expressed as the percentage.

### Histological analysis of testes

For histological studies, the transversal cuts of right testes were performed from the central part and fixed in 10 % neutral formalin solution. The pieces were dehydrated in a graded ethanol series of 70–100 % and processed for embedding in paraffin. Then, 3–5-m sections were achieved from paraffin blocks and subsequently stained with hematoxylin and eosin (H&E) and examined by light microscopy on an Olympus BX51 (Olympus Optical Co. Ltd., Tokyo, Japan). Johnsen’s score was used for spermatogenesis assessment [30]. Briefly, more than 100 cross sections of the seminiferous tubules from each animal were scored as represented in Table 1, and the mean value was calculated.

**Table 1:** Johnsen score for spermatogenesis assessment

Score	Histological criteria
10	Full spermatogenesis
9	Slightly impaired spermatogenesis, many late spermatids, disorganized epithelium
8	Less than five spermatozoa per tubule, few late spermatids
7	No spermatozoa, no late spermatids, many early spermatids
6	No spermatozoa, no late spermatids, few early spermatids
5	No spermatozoa or spermatids, many spermatocytes
4	No spermatozoa or spermatids, few spermatocytes
3	Spermatogonia only
2	No germinal cells, Sertoli cells only
1	No seminiferous epithelium

### Measurements of lipid peroxidation

The content of primary lipid peroxidation (LPO) products (diene conjugates) in serum was measured spectrophotometrically [31]. The content of thiobarbituric acid reactive substances (TBA-reactive substances) was studied by the reaction with thiobarbituric acid [32]. The concentration of Schiff bases, final lipid peroxidation products, was measured fluorometrically [33].

### Measurements of antioxidant enzyme activities

The antioxidant system state was estimated by the activities of SOD [34] and catalase [35] in serum.

### Ultrasound of the rat testes

We performed ultrasonography (US) of testes in rats using linear 5–12 MHz frequency probes of ultrasound scanner Ultrasound Philips/ATL HDI 3500 (Netherlands) before and 30 days after the intervention. We obtained transversal and longitudinal measurements and calculated volume. We used the most common formula to calculate a testicular volume for an ellipsoid structure: length (*L*) *×* width (*W*) *×* height (*H*) *×* 0.52.

### Statistical analysis

Statistical analysis performed by using the SPSS-20 software. All data in this study were expressed as means ± standard error (M ± SE) or %. Data distribution was analyzed using the Kolmogorov–Smirnov normality test. Continuous variables with parametric distribution were then analyzed using analysis of variance (ANOVA), and if the results were significant, a post hoc Tukey test was performed. For data with nonparametric distribution, Kruskall–Wallis test was used. For comparisons of categorical variables, we conducted a *χ*^2^ test. The difference between groups was defined to be statistically significant when a *p* value was less than 0.05.

### Ethics statement

This study was carried out in strict accordance with the recommendations in the Guide for the Care and Use of Laboratory Animals of the National Institutes of Health and the general ethical principles of animal experiments approved by the First National Congress on Bioethics Ukraine (September 2001). The protocol was approved by the Committee on the Ethics of Animal Experiments of the Taras Shevchenko National University of Kyiv. The rats were kept in collective cages in controlled conditions of temperature (22 ± 3 °C), light (12 h light–dark cycle), and the relative humidity (60 ± 5 %). The animals were fed laboratory chow (PurinaW) and tap water ad libitum.

## Results

### Body and reproductive organ weights

The total body weight of rats after a 10-day administration of CNPs or citrate did not differ from that of the control animals. The external examination of the male reproductive organs did not find differences between the control and both treated groups. We showed comparable similar absolute and relative reproductive organ weight in experimental and control groups, respectively. The only difference from those of the control group is a statistical tendency to increase the seminal vesicles weight in both treated groups (*p* = 0.084). The data are summarized in Table 2. Motor activity, appearance, and food and water consumption were similar in all groups. All these data indicate the absence of a toxic effect of CNPs and citrate on aging male rats in conditions of the 10-day administration.

**Table 2:** Total body weight, absolute and relative reproductive organ weights of male rats

Parameters	Control (*n* = 10)	Citrate (*n* = 10)	CNPs (*n* = 10)	*p*
Absolute weight				
Total body weight, g	396.2 ± 6.48	405.6 ± 9.26	403.5 ± 7.45	0.676
Testis, mg	2991.3 ± 98.91	2997.5 ± 107.72	3047.5 ± 70.02	0.897
Epididymis, mg	985.7 ± 28.5	970.4 ± 21.78	987.0 ± 37.74	0.910
Seminal vesicle, mg	727.1 ± 9.74	756.4 ± 10.35	754.8 ± 9.85	0.084
Ventral prostate, mg	498.02 ± 8.04	497.87 ± 7.75	492.61 ± 8.04	0.832
Relative weight				
Testis, mg/100 g	7.53 ± 0.18	7.37 ± 0.16	7.56 ± 0.18	0.730
Epididymis, mg/100 g	2.48 ± 0.05	2.39 ± 0.04	2.44 ± 0.07	0.551
Seminal vesicle, mg/100 g	1.83 ± 0.01	1.86 ± 0.03	1.86 ± 0.03	0.614
Ventral prostate, mg/100 g	1.25 ± 0.01	1.21 ± 0.01	1.22 ± 0.02	0.252

Values expressed as mean ± SEM. One-way ANOVA test was performed

### Serum sex hormone level

**Fig. 3:**
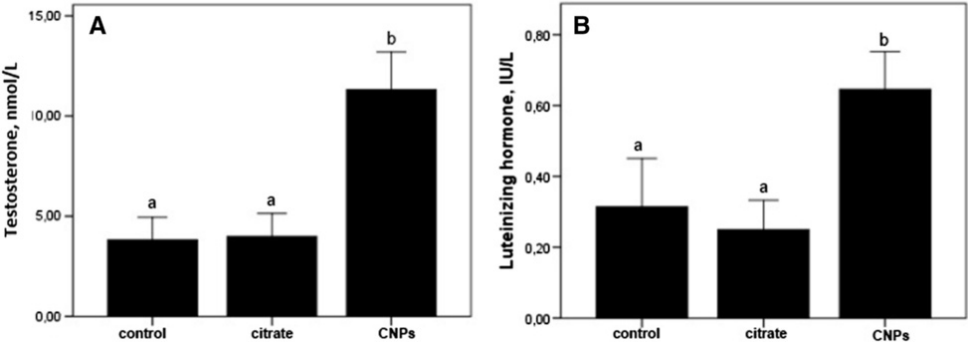
Testosterone and LH level in rats of different experimental groups. Values expressed as mean ± SEM. One-way ANOVA test followed by Tukey test was performed. **a**, **b** Mean values with the same letter do not differ statistically; values <0.05 were considered significant

Evaluation of the blood serum testosterone (Fig. 3a) showed that in the control group, the individual value ranged from 1.54 to 7.21 nmol/L (with mean value of 3.81 ± 0.56 nmol/L), which corresponded to an age-dependent decrease of hormone in male rats. Administration of CNPs was accompanied with 2.5- and 2-fold significant increase of serum testosterone and LH level, respectively, as compared with the control and citrate-treated rats (Fig. 3). Citrate administration did not lead to significant changes of testosterone (*p* = 0.985) or LH (*p* = 0.698) values when compared with the control group.

### Epididymal sperm parameters

The qualitative and quantitative sperm parameters for all groups are summarized in Table 3. Administration of CNPs at dose 1 mg/kg for 10 days resulted in significant increase of sperm count by 18.7 % (*p* < 0.001) and 16.0 % (*p* < 0.001) as compared with the control and citrate-treated groups, respectively.

**Table 3:** Epididymal sperm parameters in different experimental groups

Sperm parameters	Control (*n* = 10)	Citrate (*n* = 10)	CNPs (*n* = 10)
Concentration (10^6^/mL)	37.11 ± 1.32а	37.97 ± 1.04а	44.05 ± 1.49b
Rapid motility, % (grade A)	29.21 ± 1.3а	30.67 ± 0.72а	37.96 ± 1.5b
Slow motility, % (grade B)	25.56 ± 2.03а	25.57 ± 1.63а	29.31 ± 1.08а
Progressive motility, % (grade A + B)	54.77 ± 1.81а	56.24 ± 1.38а	67.27 ± 1.78b
Non-progressive motility, % (grade C)	25.02 ± 1.49а	23.87 ± 0.84а	17.61 ± 1.24b
Immotile sperm, % (grade D)	20.21 ± 1.58а	19.86 ± 1.11а	15.11 ± 1.02b
Viability, %	83.36 ± 1.64а	85.37 ± 1.33аb	89.3 ± 1.18b
Normal morphology, %	83.25 ± 1.28а	84.4 ± 1.22аb	88.85 ± 1.35b
Abnormalities of the sperm head, %	5.95 ± 0.57а	5.15 ± 0.57аb	3.4 ± 0.64b
Abnormalities of the flagellum, %	10.8 ± 0.87а	10.55 ± 0.9аb	7.75 ± 0.77b

Values expressed as mean ± SEM. One-way ANOVA test followed by Tukey test was performed. Mean values with the same letter do not differ statistically; *p* values <0.05 were considered significant

Our results indicated a significant improvement of sperm progressive motility (grade A + B) after CNP treatment as compared with other groups. These changes were associated with significant increase of rapid motility sperm count (grade A) by 29.5 % (*p* = 0.002) parallel with remarkable decrease of spermatozoa with nonprogressive motility (grade C) by 29.6 % (*p* = 0.001) and immotile spermatozoa (grade D) by 25.2 % (*p* = 0.022) as compared with the control. Similar changes were revealed when comparing both treated groups. But significant changes were not demonstrated between sperm motility parameters in control and citrate-treated rats (Table 3).

Sperm viability was significantly increased by 7.3 % in rats after treatment with CNPs when compared with the control group (*p* = 0.016). No significant changes were observed between both treated groups (*p* = 0.136).

In addition, the percentage of normal sperm morphology was significantly increased in rats that received CNPs as compared with the control group (*p* = 0.013) and no difference was found for citrate-treated group (*p* = 0.054). The percentages of an abnormal flagellum (coiled tail, wrong angle connection to the head) were 7.75 ± 0.77 in rats of the CNP group and were more prominent than spermatozoa head abnormalities. The percentage of spermatozoa with abnormalities of head and flagellum significantly decreased only after treatment with CNPs when compared with intact rats.

### Histological assessment of the testes

**Fig. 4:**
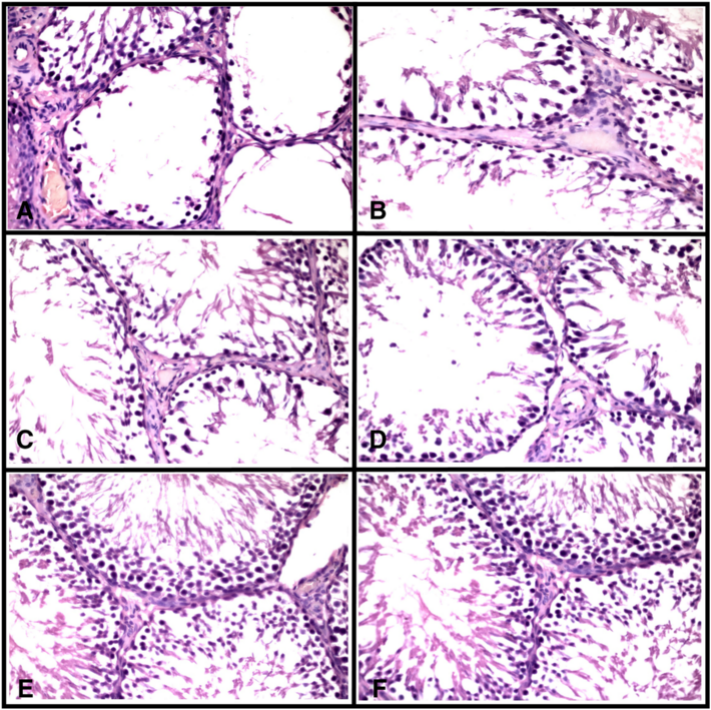
Light microscopy of cross sections of H&E stained testis. **a**, **b** Control group; **c**, **d** citrate-treated group; **e**, **f** CNP-treated group. Regular seminiferous tubules (**a**) with atrophy of spermatogenic epithelium. Irregular seminiferous tubules where spermatogenic layer is represented by focal and low amounts of cells which does not provide normal maturation of sperm; in the lumen, small amount of spermatozoa (**b**–**d**). There is a partial recovery of spermatogenic layer in the seminiferous tubules compared with the control group—observed a higher amounts of cells which are placed in several rows; the presence of spermatozoa in the lumen (**e**, **f**). Final magnification: ×400

Histological analysis has shown that the morphological structure of the testes of aging males in the control group corresponded to the overall picture of age-related changes. We indicated irregular partially regressed (contained fewer spermatogenic cells) or fully regressed (contained no spermatogenic cells) seminiferous tubules (Fig. 4a, b). The focal atrophy in the seminiferous tubules was associated with the loss of spermatogenic cells as a result in part or completely from a decreased ability of Sertoli cells to support germ cell survival and differentiation. Also in the control group, we found the number of activated Leydig cells (mean in volume and poor vacuolated cytoplasm, slightly hyperchromic, oval-shaped nuclei) decreasing.

After the 10-day treatment with CNPs, we observed the signs of regeneration in seminiferous tubules which were related to increased number and focal proliferation of spermatogenic cells (Fig. 4e, f). Also, the number of activated Leydig cells increased. Their activation indicates the presence of larger in volume and size normochromic nuclei with clear nucleoli as compared with the control. Their cytoplasm was also greater with increased number of vacuoles on the cell periphery. Activation of Leydig cells may be responsible for increasing testosterone levels in the blood plasma, which was set previously.

**Fig. 5:**
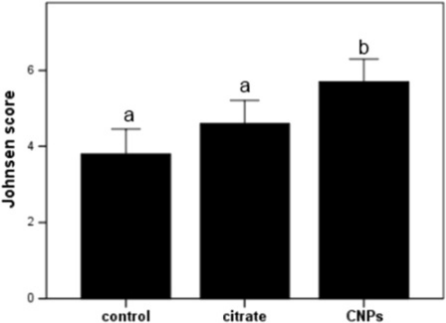
Assessment of spermatogenesis using Johnson score. Values expressed as mean ± SEM. One-way ANOVA test followed by Tukey test was performed. *a*, *b* Mean values with the same letter do not differ statistically; *p* values <0.05 were considered significant

Visual signs of a stimulation of spermatogenesis in rats that received CNPs were confirmed by Johnson score assessment. As it is shown in Fig. 5, administration of CNPs resulted in the increase of mean values by 2 and 1 point of Johnson score as compared with the control and citrate-treated rats.

Finally, we can perceive that administration of CNPs, but not citrate, slightly reduces the signs of age-related changes in testicular tissue and activated a process of spermatogenesis.

### Products of lipid peroxidation and antioxidant enzyme activities

The level of LPO products is one of the biochemical markers of oxidative–antioxidative balance. We determined the content of primary LPO products—conjugated dienes, intermediate, TBA-reactive substances, and the end LPO products—Shiff bases.

We revealed that after CNP administration, the content of LPO products in serum significantly decreased: conjugated dienes—2.1 times, TBA-reactive substances—1.5 times and Schiff bases—2.9 times as compared to the control or citrate-treated groups (Table 4). The activity of SOD and catalase was significantly increased by 1.5-fold in rats which received CNPs when compared with other groups (Table 4).

**Table 4:** Products of lipid peroxidation and antioxidant enzymes activities in different experimental groups

Parameters	Control (*n* = 10)	Citrate (*n* = 10)	CNPs (*n* = 10)
Conjugated dienes, nmol × mg protein^−1^	80.79 ± 6.02a	77.9 ± 4.3a	37.07 ± 3.27b
TBA-reactive substances, nmol × mg protein^−1^	41.22 ± 3.02a	37.94 ± 3.59a	27.06 ± 2.55b
Schiff bases, units × mg protein^−1^	5.61 ± 0.51a	5.26 ± 0.4a	1.82 ± 0.54b
SOD, units × min^−1^ × mg protein^−1^	0.11 ± 0.01a	0.11 ± 0.01a	0.16 ± 0.01b
Catalase, nmol × min^−1^ × mg protein^−1^	0.44 ± 0.04a	0.45 ± 0.04a	0.63 ± 0.05b

Values expressed as mean ± SEM. One-way analysis of variance (ANOVA) test followed by Tukey test was performed. Mean values with the same letter do not differ statistically; *p* values <0.05 were considered significant

### Ultrasound assessment of testes

We obtained a slight increase of linear measurements of testes in the CNP group as follows: 11 ± 1.1 mm × 11 ± 1.3 mm × 15 ± 1.5 mm on baseline before CNP introduction up to 12 ± 1.2 mm × 12 ± 1.3 mm × 17 ± 1.6 mm on the 30th day of experiment (*p* > 0.05). In controls, linear measurements of testes were on the 30th day of experiment 11 ± 1.1 mm × 10.8 ± 1.4 mm × 15 ± 1.5 mm, and in the citrate group, 11 ± 1.1 mm × 11 ± 1.2 mm × 13 ± 1.6 mm, respectively.

**Fig. 6:**
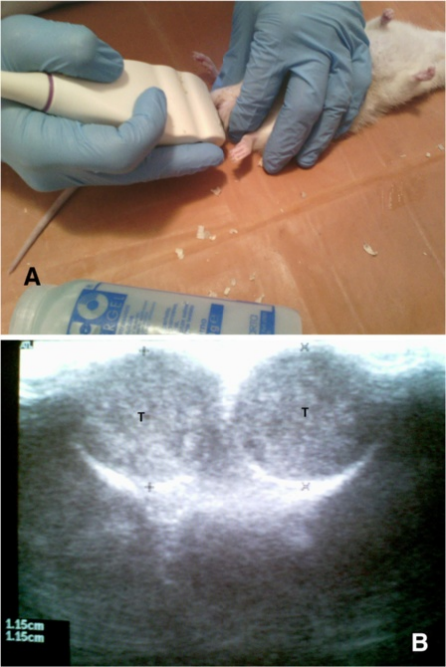
Ultrasound assessment of testes in rats. **a** View of ultrasound technique to obtain transversal scans of testes in rat. **b** Transversal US scans of testes (*T*) and linear measurements

Volume changes were calculated as 0.943 ± 0.44 ml on baseline vs 1.272 ± 0.5 ml (*p* > 0.05) in the CNP group, and insignificant decrease to 0.817 ± 0.42 was calculated for the citrate group (*p* > 0.1). Changes in the echo structure of testes were not registered (Fig. 6).

## Discussion

There are several factors which could influence the CNP catalytic activity, as well as their antioxidant or pro-oxidant biological response. Tissue environmental conditions such as pH play an important role in the determination of CNP direct oxidant behavior [36, 37]. In a neutral pH environment, CNPs act as antioxidant that is cytoprotective agent [38]. Conversely, in pancreatic cancer cells (L3.6pl) with acidic pH, pretreatment with CNPs revealed a twofold increase in ROS after 30 min and up to 24 h of postradiation exposure. In normal CNP-treated pancreatic cells, there was a 50 % decrease in ROS [39]. In pH-dependent pro-oxidant effect conditioned by inhibition of catalase activity [40] in cancer cells, CNPs are only capable of catalyzing the conversion of highly unstable superoxide to far more stable H_2_O_2_. Without catalase mimetic ability to remove H_2_O_2_, CNPs actually increased the toxicity of radiotherapy in cancer cells due to an accumulation of ROS in the cell [39].

Synthesis methods and physiochemical properties (size, shape, agglomeration status in liquid and coating) of CNPs could also influence their biological response and catalytic activity. Several nanocomposites of CNPs used for coating biocompatible polymers (polyethylene glycol, dextran, polyacrylic acid, citric and oleic acid) were described [8]. Coating of nanoparticles with a surfactant or biocompatible polymers improves stability, increases dispersion and blood circulation time, decreases nonspecific interactions with cells and proteins, and reduced toxicity [8, 41]. In our study, we used near-shaped, citrate-coated CNPs with particle size 2–5 nm. Ammonium citrate in nanocomposites serves not only as a stabilizer, which determines the compositional stability of CNPs in water and biologically relevant media, but also plays an important role in the pharmacokinetics of CNPs in the cells and established that ammonium citrate formed a multinuclear complex with CNPs leading to the formation of monodisperse, uniform in size (2–5 nm) particles. Additionally, citrate provides a negative zeta potential of CNPs that are less toxic to normal mammalian cells. Finally, citrate as a compound in Krebs cycle provides CNPs to the mitochondria where under the pathologic conditions ROS forms leading to oxidative stress. Therefore, the CNP antioxidant function appears in the zone of maximum ROS concentration. In our study for distinguishing biological response of both nanocomposite components, we used sodium citrate as active comparator to CNPs in the same dose and course.

Most prominent factors associated with age-related decrease of spermatogenesis and steroidogenesis are dysregulation of the hypothalamus–pituitary axis, reduction of *Leydig* cell count, which produced more than 60 % of androgens, lower density of LH receptors in the cells, and decrease in testosterone synthesis enzyme activity. Also, it has been shown that apoptosis increases with age producing an accelerated germ cell loss as a result of oxidative stress in the tissue [42].

ROS are defined as molecules with one or more unpaired electrons in their outer orbital. Due to the presence of unpaired electrons, these molecules are very unstable and tend to react with neighboring molecules, depriving them of electrons and thus altering their structure and function. ROS can damage cells by starting chemical chain reactions such as lipid peroxidation or by oxidizing DNA or proteins [43].

ROS can exhibit a dual effect on the male reproductive system. In a healthy organism, small amounts of ROS are produced by spermatozoa and they play an important role as key signal molecules which are involved in capacitation and acrosome reaction [17, 18]. The balance between their production and inactivation by various scavengers is strongly controlled due to physiological conditions [19]. Violation of this balance occurs with aging. ROS level is positively correlated with the proportion of sperm with amorphous heads, damaged acrosomes, midpiece defects, cytoplasmic droplets, and tail defects [44]. Excessive ROS formation leads to peroxidation of phospholipids in the spermatozoa mitochondria and thus impairs their motility [45] or can damage the sperm membrane and flagellum structure and disrupt morphology [23]. Oxidative stress within a sperm results in protein or DNA damage that leads to a reduction in Leydig cells and synthesis of testosterone [46]. This oxidative stress-induced sperm damage has been suggested to be a significant contributing factor in 30–80 % of all cases of male infertility [47].

CNPs due to self-regenerative ROS scavenging property have been used in different areas of medicine where pathologies are associated with excessive oxidative stress. Therefore, we aimed in this study to investigate the effect of CNPs on qualitative and quantitative sperm parameters, spermatogenesis, sex hormone levels, and pro/antioxidant state in 24-month male rats. We revealed that after 10 days, administration of CNP level of LPO in serum significantly decreased by 1.5–2.9 times, parallel with 1.5-fold increase of catalase and SOD activity. These changes of oxidative–antioxidative balance were associated with increase of sperm count by 18.7 % (*p* < 0.001) and improvement in quantitative sperm parameters such as motility, viability, and percentage of spermatozoa with normal morphology in rats that received CNPs as compared with the control group. We indicated an increase of rapid motility sperm count (grade A) by reducing spermatozoa with nonprogressive motility (grade C) and immotile spermatozoa (grade D). Significant changes within sperm quantitative parameters in control and citrate-treated rats were not designated.

We have mentioned that aging-related factors decrease the number of activated *Leydig* cells and focal atrophy of the seminiferous tubules which are often adjacent to tubules exhibiting normal spermatogenesis. These data are in agreement with previously reported studies [48, 49].

After the 10-day treatment with CNPs, but not with citrate, we observed signs of regeneration in the seminiferous tubules. Visual signs of spermatogenesis stimulation were confirmed by 2-point increase of mean values of Johnson score after CNP administration as compared with the control rats.

Activation of Leydig cells may trigger a 2.5-fold significant increase of serum testosterone. Citrate administration did not lead to significant changes of testosterone (*p* = 0.985) or LH (*p* = 0.698) values when compared with the control group.

The determination of organ weights is an important parameter for assessing the risk of toxicity on the male reproductive system [50]. In our experiment, we have measured comparatively similar absolute and relative reproductive organs weights data in all groups, and testes sizes were visualized in vivo on ultrasound which indicates the absence of a toxic effect of CNPs and citrate on aging male rats in conditions of the 10-day administration.

### Toxicity of cerium oxide nanoparticles

The toxicity of cerium oxide (CeO_2_) nanoparticles (NPs) was reported as low [1–16]; however, in several issues (e.g., in regard to reproduction, potential environmental contamination, etc.), it still remains largely unknown.

The internalization and subcellular localization of nanoceria plays a key role in the nanoparticles’ cytotoxicity profile, exhibiting significant toxicity when they localize into the lysosomes of the cancer cells. In contrast, minimal toxicity is observed when they localize into the cytoplasm or do not enter the cells. Taken together, these results indicate that the differential surface charge-dependent localization of nanoceria in normal and cancer cells plays a critical role in the nanoparticles’ toxicity profile [37]. In our study, we used coating of nanoparticles with the surfactant or biocompatible polymers that improve stability, increase dispersion and blood circulation time, decrease nonspecific interactions with cells and proteins, and reduce toxicity.

Tourinho et al. concluded in their study [51] that CeO_2_ NPs have a low toxicity and do not affect a toxicity of phenanthrene to isopods and springtails. The data of the study by Lahive et al. suggest that CeO_2_ NPs do not affect survival or reproduction in earthworm *E. fetida* over the standard test period. However, there were histological changes that could indicate possible deleterious effects over longer-term exposures [52].

CNPs can protect the gastrointestinal epithelium against radiation-induced damage both by acting as free radical scavengers and by increasing the production of superoxide dismutase 2 (SOD2) before radiation insult [53]. We recently reported effects of CeO_2_ nanoparticles affecting gastrointestinal motility on rat model and reviewed data supporting their perspectives to be applied as effective laxatives [54].

A detailed investigation on the agglomeration of oxide nanoparticles in wastewater streams revealed a high stabilization of the particles against clearance [55]. The current available data do not suggest an immediate risk from acute exposures to nanoceria from use as a *fuel additive* or mechanical/chemical polishing or planarization. However, the data gaps should be addressed before a comprehensive ecological risk assessment can be performed for ceria for chronic exposures or for other exposure pathways [56, 57].

### Translation to human men health within the concept of PPPM

In recent decades, infertility has impacted an increasing number of couples. The tasks of PPPM are to develop well-balanced family life through all life spans in aging society and promote sustainable reproduction health and new healthy generation [58].

For effective translation to a human organism, we should consider a number of factors. Human infertility depends on many psychological, medical, and social aspects and is manifested by symptoms that are similar to those on the rat model mostly in a laboratory scale. Lifestyle, collateral diseases, psycho-emotional status, and related conditions should be assessed carefully in human studies to suggest integrated programs facing the problem.

### Social aspects

In 2006, approximately 10 % of couples in the United States are defined as infertile based on the inability to conceive after 12 months of unprotected intercourse [59, 60]. In the United States since 1980, the birth rate in women older than age 35 years increased by nearly 60 %, whereas the birth rate for women aged 20 to 34 years increased by only 10 %.

This trend, in addition to an age-related decline in fertility, has led to an increased need for advanced reproductive technology for couples.

*The trend for parenthood at an older age has also been seen in men*. Since 1980, the fertility rate for men in their 30s has increased by 21 % and for men aged ≥40 years, the rate has increased nearly 30 %. In contrast, the fertility rate in men younger than age 30 years has decreased by 15 % [61].

In recent years, these tendencies were kept. Thus, according to the report on data births in the United States in 2013 [62], the fertility rate of men was 45.8 births per 1000 men aged 15–54, down 1 % from 2012. Similar to fertility rates among women, rates declined for all men under age 30 (down 11 % for ages 15–19, 4 % for ages 20–24, and 2 % for ages 25–29); rose for men aged 35–54 (up 1–2 % for age groups 35–39, 40–44, and 45–49, and 4 % for ages 50–54); and were unchanged for men aged 30–34 and 55 and over. Rates for men aged 15–19 (12.3), 20–24 (55.7), and 25–29 (90.6) were again at record lows in 2013, whereas the rates for men aged 35–39 (66.6), 40–44 (27.0), and 45–49 (8.8) were the lowest in more than 40 years (16). Birth rates declined less than 1 % for both white and black men in 2013 from 2012, to 43.8 and 58.0 births per 1,000 men aged 15–54, respectively. Information on the age of the father is often missing on birth certificates of children born to women under age 25 and to unmarried women. In 2013, the age of the father was not reported for 13 % of all births, 32 % of births to all women under age 20, and 29 % of all nonmarital births. Birth rates declined for women in their 20s and increased for most age groups of women aged 30 and over. The total fertility rate declined 1 % [62].

The idea that *robust fertility for a man will continue well past a woman’s decline in fertility is untrue* [59, 61, 62].

In addition to the increased rates of obesity and medical illness found in reproductive-aged women, intentionally delayed childbearing is a trend among professional couples [63]. Thus, university students plan to have children at ages when female fertility is decreased without being sufficiently aware of the age-related decline in fertility. This increases the risk of involuntary infertility in this group, which is alarming in view of the great importance they put on parenthood [63]. On the other hand, there has been recent evidence of increased rate of *first trimester spontaneous abortion* with older paternal age. For paternal age ≥35 years, the risk of spontaneous abortion between 6 and 20 weeks of gestation was 1.27. This elevated risk was seen even when evaluating only those couples where the maternal age was <30 years. When divided by trimester, the risk of first trimester miscarriage was 1.56 and the risk was 0.87 for second trimester loss [64].

### Sexual life and fertility

Decreased coital frequency with age is due in part to diminished sexual functioning; however, sexual dysfunction itself has no known influence on germ cells and its impact on infertility can be overcome by measures of assisted reproductive technology [59, 65].

Studies have consistently shown that increasing male age is associated with an increased time to pregnancy and decreased pregnancy rates. However, only a few studies have examined these outcomes while adjusting for female age. Ford et al. performed Avon Longitudinal Study of Pregnancy and Childhood, a large population-based study in the United Kingdom. Surveys from 8559 pregnancies were used to determine the effect of age on time to pregnancy. After adjusting for female age, conception during a 12-month period was 30 % less likely for men over age 40 years as compared with men younger than age 30 years [66]. In addition to female age, coital frequency, and sexual functioning are variables that affect the time to conception and pregnancy rates. Decreased sexual activity can decrease the chances of conception, and *erectile dysfunction* (ED) increases with age [67].

### Molecular biomarkers in semen parameters

*Male factor infertility* is a term that encompasses a host of different conditions relating to sperm function that may make it difficult for a sperm to fertilize an egg under normal conditions [59].

Problems within male factor fertility may be due to changes in semen quality as assessed by the semen plasma analysis [59, 68–70]. Seminal plasma is a promising biological fluid to use for noninvasive clinical diagnostics of male reproductive system disorders to verify a list of prospective male infertility biomarkers [68].

*Molecular biomarkers* hold promise to advance the noninvasive diagnosis of male reproductive system disorders and facilitate the identification and management of these conditions through screening, early diagnosis, and more accurate prognosis.

Seminal plasma proteins arise from secretions from seminal vesicles (~65 % of semen volume), prostate (~25 %), testis and epididymis (~10 %), and bulbourethral and periurethral glands and has great potential as a proximal fluid for protein biomarker discovery and as a clinical sample for noninvasive diagnostics [60]. The most significant changes are a low sperm concentration (oligospermia), poor sperm motility (asthenospermia), and abnormal sperm morphology (teratospermia) [51]. Collection of fluids secreted by individual glands provides samples enriched with tissue-specific proteins. For example, expressed prostatic secretions can be obtained in prostate massage, whereas epididymal and seminal vesicle fluids are collected by microsurgical epididymal sperm aspiration and seminal vesicle fluid by *transrectal ultrasonography-guided aspiration* [69].

Evidence consistently indicates that sperm motility in contrast to concentration decreases with advancing age. Studies that adjusted for the duration of abstinence revealed statistically significant decreases in motility by 0.17 to 0.6 % decrease per year of age [70].

*Testis biopsy* as a variant of a variety of surgical sperm retrieval procedures can be performed for the purpose of subsequent or simultaneous IVF/intracytoplasmic sperm injection. The decision regarding the type of procedure should be based on the type of azoospermia, specific clinical circumstances, as well as on the surgeon’s preference and experience [71].

Rolland et al. [72] performed a proteomic analysis of the human seminal plasma and identified 699 proteins. By comparing the protein list with other previous proteomic data sets, they found that 2545 unique proteins have been described so far in the human seminal plasma. Authors profiled their expression at the gene level and identified 83 testicular, 42 epididymis, 7 seminal vesicle, and 17 prostate candidate protein markers. For a subset of testis-specific candidates, i.e., TKTL1, LDHC, and PGK2, their germ cell expression was validated and it was demonstrated that such markers could be distinguished between semen from fertile and infertile men.

Several promising biomarkers for male infertility and prostate cancer include ECM1, TEX101, LDHC, TKTL and ACPP proteins [68, 69, 72, 73], and prostate specific antigen (PSA), TMPRSS2-ETS [74].

The identification of genetic factors is important for appropriate management of the infertile couple. In the review by Ferlin et al. [75], 56 genes were mentioned as responsible for male reproduction (ADAM2; ADAM20; AKAP3; BAX; BCL2L10; BMP15; BRD2; C15ORF2; C8ORF1; CNA1; CCT6B; CSNK2A2; CYLC1; D8S2298E; DAZ; DAZL; DBY; FLJ13057; LJ20080; FOXJ1; FSHB; FSHPRH1; HSF2BP; HYAL4; LOC56926; MAGOH; MAK; MCSP; MOV10L1; NR6A1; ODF1; OR7C1; OVOL1; PCMF; PIWIL1; PPAP2A; PRKAG1; PRM1; PRM2; PROL1; RFP; SECP43; SMCY; SPAG1; SPAM1; SPINLW1; STK22B; STK22C; STRBP; STRIN; SYCP1; TCFL5; TESK2; TNP1; TXNDC2; ZP20) and 36 genes for spermatogenesis (BCL2L10; BRD2; C15ORF2; CCNA1; CCT6B; CSNK2A2; CYLC1; DAZ; DAZL; DBY; FOXJ1; FSHPRH1; HSF2BP; MAK; MOV10L1; NR6A1; ODF1; OR7C1; OVOL1; PIWIL1; PRKAG1; PRM1; PRM2; PROL1; RFP; SECP43; SMCY; SPINLW1; STK22B; STK22C; STRBP; SYCP1; TCFL5; TESK2; TNP1; TXNDC2). Several human genes are known with mutations causing male infertility (AR; AZF gene families; CFTR, DM-1, DNAH gene family, FGFR1, FSHR, INSL3, KAL-1, LGR8-GREAT, LHR, POLG) [76].

The specific seminal markers investigated were glucosidase secreted by the epididymis, PSA and zinc secreted from the prostate, and fructose secreted by seminal vesicles. Glucosidase, PSA, zinc, and fructose levels are significantly lower in men aged over 50 years compared with men aged between 21 and 30 years [77].

Collection of the large list of identified biomarkers for a rapid diagnostic tool for the short- and long-term monitoring of pathological disorders and drug therapy is a promising challenge for screening. For instance, the collection of saliva, either any of biological fluids of human body, that is a fairly easy and noninvasive procedure and not safe to the patients, has a strong potential for predictive screening programs [78].

The potential development of electronic tools for self-monitoring via mobile gadgets would be a very attractive solution of high-tech PPP medicine.

### Oxidative stress and nitrosative stress biomarkers for male infertility screening and pregnancy planning

Nanoceria particle impact on oxidative stress which includes oxidative stress and nitrosative stress biomarkers for male infertility screening and pregnancy planning is essential. These are as follows:

- *Oxidative stress* biomarkers. DNA oxidation biomarkers (oxidized DNA bases such as 8-OHdG, autoantibodies to oxidized DNA, modified comet assay), lipid oxidation (thiobarbituric acid-reactive substances, exhaled pentane/ethane, low-density lipoprotein resistance to oxidation, isoprostanes), protein oxidation (protein carbonyls);
- *Nitrosative stress* biomarkers. NO, nitrite, peroxynitrite, and inducible NO synthase (iNOS) expression, modificated proteins, nitrosothiols, 8-nitroguanine (a marker of nitrative DNA damage), etc. [79, 80].

As nanoceria were reported to impact on fertility [27] and to have wide antioxidative properties in different tissues [81], the profound study of the mechanism of its action might be important to understanding some particular genesis of infertility.

### Apoptosis, infection

Sperm apoptosis is related to male age, body mass index (BMI), testicular volume, and FSH. Among the apoptotic markers, only DNA denaturation has been found to predict natural pregnancy better than conventional sperm parameters [82, 83]. Thus, DNA denaturation correlated positively with age and negatively with testicular volume (TV). MMP correlated negatively with BMI and FSH and positively with TV. Normal viable sperm correlated positively with TV and negatively with age, BMI, and FSH. DNA denaturation was associated with a significantly lower natural pregnancy rate.

For men, viral orchitis and sexually transmitted infections can lead to infertility due to germinal cell damage, ischemia, or the immune response to the infection. Temporary inflammatory episodes in the male reproductive tract which are self-limiting are probably common [84].

The risk of developing a medical condition or of being exposed to environmental toxins increases with age, e.g., accumulation of the aging pigment *lipofuscin* increasing with age, which decline Leydig cell and Sertoli cell numbers [59].

### Ultrasound

Other factors less well associated with infertility include semen volume and other seminal markers of epididymal, prostatic, and seminal vesicle function. As men age, these variables are impacted and correlate with decreased fertility [59].

*Ultrasonography* (*ultrasound, US*) is a widely used and well-tolerated imaging modality for evaluation of pathologic conditions of the testes. Recent technical advances of US applications and postprocessing developments have enabled new aspects in the structural and functional analysis of testicular tissue and, therefore, male fertility [85–87].

**Fig. 7:**
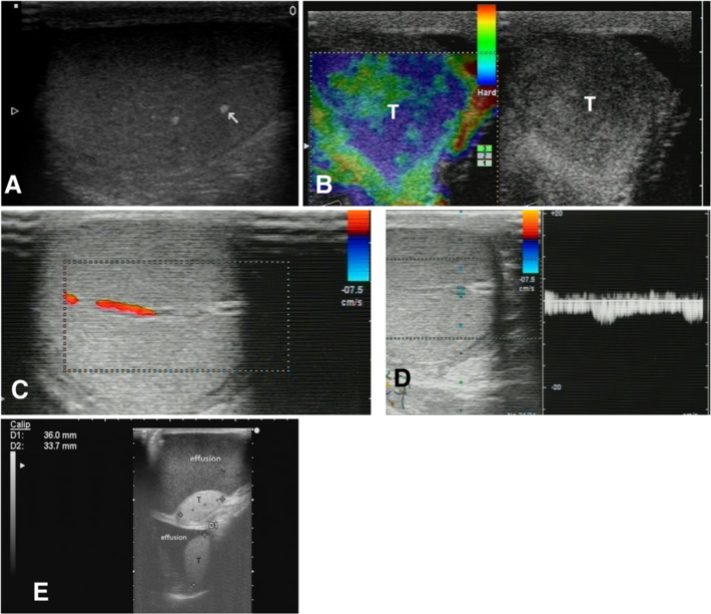
Ultrasound assessment of testes in infertile men—imaging biomarkers. **a** Testicular microlithiasis (*arrow*). **b** Testicular tumor—seminoma (T), tumor is better visualized using sonoelastography (performed on Hitachi US equipment) that depicts stiff lesion in the parenchyma (colored as *blue* on the left side of the figure). **c** Testicle perfusion—Doppler. **d** Doppler spectrum of artery in testis. **e** Hydrocele—on US scan are visualized testicles (*T*) and effusion around both testicles

Testicular ultrasound should be performed in every patient with unexplained infertility and abnormal sperm analysis. It allows diagnosis of more pathologic conditions than physical examination (Fig. 7). The obtained results demonstrating the increase of the rat testes volume on in vivo ultrasound exceeding morphological size measurement were most likely due to blood perfusion in tests in living animals’ tissues.

Several ultrasound scans of clinical observations of infertile men are presented in Fig. 7 (examinations were performed in Clinical Hospital “Pheophania” in Kyiv, Ukraine, using ultrasound equipment Hitachi HV900 and BK “Hawk”).

Besides a rapid varicocele screening, color Doppler ultrasound allows us to evaluate its hemodynamic repercussion by studying the spectral display, color, and response to Valsalva’s. It also provides an exact measure of testicular volume and allows to detect the presence of dystrophic changes in the testicle, as well as anomalies of the epididymis and vas deferens, such as cystic dilations. It is also the test of choice to detect undescended testicles. Ultrasound may also detect nonpalpable testicular tumors (Fig. 7b) which are more prevalent in this group of patients [86].

*Testicular size* may be a surrogate marker of spermatogenesis [88]. The size of the testis is almost unchanged until the eighth decade, at which point the testicular volume was reported to be 31 % lower than in men aged 18 to 40 years [89]. The testes are responsible for the production of spermatozoa and testosterone in men. Approximately 80–90 % of the testicular volume is made up of seminiferous tubules and germ cells [59]. Thus, a reduction in the number of these cells is manifested in a reduction in testicular volume [88, 89].

Studies in infertile men have shown that the testicular volume has a direct correlation to seminal fluid and sex hormone assay, just like the simple measurement of testicular length, width, and depth [90].

The testicular volume can be calculated using (a) the formula for an ellipsoid (formula 1): length (*L*) *×* width (*W*) *×* height (*H*) *×* 0.52; (b) the formula for a prolate spheroid (formula 2): *L* × *W*2 × 0.52; and (c) the empiric formula of Lambert (formula 3): *L × W × H ×* 0.71 [91].

Testicular microlithiasis (TM) can be revealed on testicular ultrasounds (Fig. 7a) in men suffering from the conditions such as scrotal pain, swelling, and infertility [92].

In addition, evidence exists that testicular perfusion assessed by routine color Doppler ultrasonography is valuable for diagnosing scrotal abnormalities in infertile men, frequently detecting nonpalpable lesions [93, 94]. Increased resistive index (RI) and pulsatility index (PI) of capsular branches of testicular arteries on unenhanced color Doppler US examination may be an indicator of impaired testicular microcirculation.

*Contrast-enhanced ultrasound* (CEUS) imaging is potentially applicable to the investigation of vascular disorders of the testes providing additional information regarding testes vascularity [86].

Evidence suggests that there is a mild decrease in seminal volume with increasing age, although the clinical significance of this finding is marginal. The decrease in volume may be related to seminal vesicle insufficiency because seminal vesicle fluid composes most of the ejaculate volume [59].

Prostatic changes, including smooth muscle atrophy, may also affect semen volume and sperm motility. Complex use of radiological methods for prostate including magnetic resonance imaging (MRI), magnetic resonance (MR) spectroscopy, positron emission tomography (PET), Doppler, sonoelastography, transrectal US-guided prostate biopsy, and scrotal ultrasound provides significant data to assess the health of men with infertility-associated cases [92–96].

*Transrectal ultrasonography*-guided seminal vesicle fluid aspiration biopsy is a safe and effective technique to collect material for molecular/genetic biomarkers associated with spermatogenesis in infertile men [96].

*Microdialysis needle* might provide longitudinal in vivo assessment of molecular tissue biomarkers, and the potential to provide immediate feedback-guided treatment and the relevant research in the field [97], e.g., tissue cadmium level, calcium channel sequence, and other markers, may predict the outcome of varicocele surgery [97].

Existing data suggest that there are wide geographical variations in semen quality and in the incidence of *testicular cancer*, *cryptorchidism*, and *hypospadias* and emphasize the importance of the *environmental* antiandrogenic compounds for the development of the testis and reproductive tract. The data illustrate the similarities between the effects of some environmental compounds (as phthalate esters) and perceived decline in human male reproductive health which comprises *testicular dysgenesis syndrome (TDS)* [98]. Accumulation of toxic agents including the products of cigarette smoke such as *cadmium* was reported to be elevated in seminal plasma from infertile men with varicocele (compared with those without varicocele) [99].

*Varicocele* and *hernia* are the most common risk factors in men attending the infertility. In addition, *strenuous work* could cause testicular injury [100]. Increased apoptosis among developing germ cells was demonstrated, which may be the cause of oligospermia and testicular tissue of men with varicoceles. The increased levels of ROS were shown in association with poor sperm motility; morphologically abnormal spermatozoa have demonstrated disruption of actin in the sperm heads by cadmium, and its cations were reported to be present in high concentrations among some men with varicoceles [99–101]. There was no evidence that antisperm antibodies are responsible for the damage to spermatogenesis in unilateral cases; however, measurement of the serum hormones showed elevations of LH and FSH which cannot be entirely explained as secondary to the damaged spermatogenesis. There was weak but nevertheless significant evidence that *testis maldescent* may be a poor prognostic factor as far as subsequent fertility [101, 102].

Patients demonstrating *congestive* phenomena with predominance in the pelvis and increased temperature in testes is a large group, who should be stratified in/subfertility management. Interactions between skeletal muscle spasticity, myofascial pain pelvic pain, and pelvic floor muscle dysfunction [103] and also with microcirculation disorders due to vasospasm like *Flammer syndrome* [104] might give intriguing pathogenesis clues in understanding this psyche–intestine–circulation–pain–movement–hormonal interaction as a whole.

Mohr et al. recently conducted the cohort controlled study for confounding factors such as chronic illness, BMI, medications, and lifestyle when analyzing testosterone levels. They reported that chronic disease and high BMI significantly decreased testosterone concentrations, whereas smoking tended to increase a total, free, and bioavailable testosterone concentration. Finally, declining testosterone may cause a decline in libido, erectile dysfunction, and difficulty achieving ejaculation [105].

### Metabolic syndrome

Metabolic syndrome (MetS) is a compilation of symptoms including central obesity, insulin resistance, dyslipidemia, hypertension, glucose intolerance, insulin resistance, and hypertension and comprises an array of cardiovascular disease (CVD) risk factors [106–109]. Currently, there is sufficient evidence to suggest a MetS–male infertility paradigm. Obesity/overweight may result in hypogonadism, increased scrotal temperatures, impaired spermatogenesis, decreased sperm concentration and motility, and increased sperm DNA damage. Similarly, noninsulin-dependent diabetes mellitus (NIDDM)/insulin resistance may contribute to and compound this scenario. Dyslipidemia with increased oxidative stress in the testicular microenvironment and/or an excurrent ductal system may further decrease fertility [106, 107].

Currently, there are limited prospective studies examining the effects of treating metabolic syndrome on male reproduction, and these relationships will need to be a focus of further investigation [106, 107]; additional studies are needed to fully elucidate the pathophysiologic link between the components of MetS and male infertility.

Retrospective analysis of the clinical, laboratory (spermatic, microbiological, and hormonal), ultrasonography integrated data led on a *diabetic* population with the vascular extra-genital pathology confirms data relative to the heterogeneity of the clinic-andrological presentation of the diabetic patient [109, 110]. Diabetes is associated with increased sperm nuclear and mtDNA damage that may impair the reproductive capability of men [111].

The links of neurodegenerative diseases (NDD) with male infertility is not still supported by solid evidence.

However, a large proportion of infertile males are still diagnosed as idiopathic, reflecting poor understanding of the basic mechanisms regulating spermatogenesis and sperm function [76].

### Male in/subfertility and erectile dysfunction treatment approaches

There are few commonly accepted treatments for male subfertility [112–114]. *Antioxidative agents are in focus for search of new treatment modalities*, as the excessive generation of reactive oxygen species by abnormal spermatozoa and leukocytospermia has been identified as one of the few defined etiologies for male infertility. Seminal oxidative stress in the male reproductive tract is known to result in peroxidative damage of the sperm plasma membrane and loss of its DNA integrity. Normally, a balance exists between concentrations of reactive oxygen species and antioxidant scavenging systems. However, during genitourinary infection/inflammation, these antioxidant mechanisms may downplay and create a situation called oxidative stress. Limited endogenous mechanisms exist to reverse these damages. In a normal situation, the seminal plasma contains antioxidant mechanisms which are likely to quench these ROS and protect against any likely damage to spermatozoa.

One of the rational strategies to counteract the oxidative stress is to increase the scavenging capacity of seminal plasma.

*Numerous studies have evaluated the efficacy of antioxidants in male infertility* [22, 106, 113–115]*.*

Therefore, *antioxidants* such as vitamin C, vitamin E, glutathione, and coenzyme Q10 have proven beneficial effects in treating male infertility. A multifaceted therapeutic approach to improve male fertility involves identifying harmful environmental and occupational risk factors while correcting underlying nutritional imbalances to encourage optimal sperm production and function [113–115].

Conducted quantitative meta-analysis improves precision where individual trials are not powerful enough to demonstrate moderate treatment effects. The quality of most trials is poor. Although many clinical trials have demonstrated the beneficial effects of antioxidants in selected cases of male infertility, some studies failed to demonstrate the same benefit. The majority of the studies suffer from a lack of placebo-controlled, double-blind design, making it difficult to reach a definite conclusion. In addition, investigators have used different antioxidants in different combinations and dosages for varying durations.

Pregnancy, the most relevant outcome parameter of fertility, was reported only in a few studies, and few conventional treatments (i.e., those not involving assisted conception techniques) result in improved fertility rates [112].

Most studies failed to examine the effect of antioxidants on a specific group of infertile patients with high oxidative stress. Multicenter, double-blind studies with statistically accepted sample size are still needed to provide conclusive evidence on the benefit of antioxidants as a treatment modality for patients with male infertility.

*Thus, so far, the studies which assessed therapies of infertile men with potential treatment agents found no improvement in sperm parameters after treatment* [112–115].

### Diet

Development of person-oriented diets is in the focus of PPPM which have a beneficial impact on many aspects of health [116, 117] and male fertility [118, 119]. Despite the fact that precise mechanisms have yet to be established, there is a significant role for both diet and physical activity to improve the many factors associated with the metabolic syndrome, including modulation of various *adipocytokines* [107–109].

Popular dietary supplements like monosodium glutamate were reported to evoke a toxic effect on testicular structure in rats, decrease testicular weight, decrease tubular diameter, reduce germinal epithelium height, decrease the spermatic count, and cause abnormalities of sperm morphology [120]. The beneficial effects of diet correction including probiotics on glutamate-induced metabolic disorders were demonstrated [117].

Although soy-based food consumption has been frequently associated with beneficial health effects, it was reported to include increased levels of phytoestrogens (plant estrogen) and, endocrine-disrupting chemicals (EDCs), having potential adverse effects on the reproductive and endocrine systems, and fertility. Soybean is the most important dietary source of *isoflavones*, an important class of phytoestrogen. The intake of isoflavone can impact semen quality parameters and induce infertility among men [121]. The phytoestrogens genistein and vinclozolin, present in soy products, may increase risks of the incidence of hypospadias up to 41 % with consumption of genistein and vinclozolin together [122].

Thus, the *vegetarian* diet style that includes soy-based foods might be associated with a higher risk on the male reproductive male system.

In a study of the likely risk factors, such as smoking and drinking habits, *smoking* and the density and viability of sperm were suggested to be significant predictors of male infertility [123].

### Sport, physical activity

However, there is a little evidence that exercise training induces male infertility; no evidence exists regarding the exact pathophysiological mechanism operational in the exercise-induced changes in the reproductive hormones [124]. The inclusion of exercise training as a component of male factor infertility has been proposed secondary to changes observed in the reproductive hormone and semen profile of some endurance-trained male athletes.

Controversial data exist regarding the effects of physical activity on fertility, with the prevalence of trials demonstrating worsening of seminal parameters. Thus, a subset of endurance-trained men, particularly runners, present with subclinical changes in their reproductive hormone profile [125]. Some activity is unfavorable for fertility as, e.g., mechanical trauma to the testes associated with cycling and/or increased gonadal temperature; however, it has not been suggested that endurance exercise adversely affect the *hypothalamic–pituitary–testis axis* [126], strenuous work could cause testicular injury [100]. It has been demonstrated that physical stress in healthy male athletes can interfere with LH levels [127].

Bicycling was suggested to cause the prolonged compression of perineal arteries leading to reduced chronic penile perfusion inducing impotence [127], with an incidence of erectile dysfunction 13–24 %. High frequency of lower urinary tract symptoms (LUTS) in cyclists has been related to increase the incidence of erectile dysfunction in comparison with a normal population.

A high incidence (up to 30 %) of varicocele has been reported in a population of athletes and up to 60–80 % in the subgroup of body builders. The incidence of varicocele specifically increases with hours of training, in a linear model [127].

According to a randomized controlled study by Safarinejad et al. [128], long-term strenuous treadmill exercises (overtraining syndrome) have a deleterious effect on reproduction.

Unclear evidence exists on the effects of *radiofrequency fields* on human reproductive health [129–131]. Possible relationship was demonstrated between exposure to radiofrequency fields during work with radar and reduced fertility [130]. Radiofrequency electromagnetic waves emitted from cell phones may lead to oxidative stress in human semen [131]; thus, keeping the cell phone in a trouser pocket in talk mode may negatively affect spermatozoa and impair male fertility.

### Infertility-related PPPM-oriented questionnaires for men health

Development, validation, and implementation of linguistically validated evidence-supported PPPM-oriented questionnaires for men health questionnaires are essential to assessment conditions specific to infertility which is tailored to the person. A number of male health questionnaires are successfully used, such as Male Sexual Health Questionnaire (MSHQ) [130], International Prostate Symptom Score (IPSS) [132], Male Fertility Evaluation Questionnaire by Dr. Schlegel [133], etc. International Index of Erectile Function (IIEF) scores that are used for assessment ED do not statistically differentiate among the specific etiologies of ED as determined by evidence-based testing with color duplex Doppler ultrasound testing [134]. On the other hand, the IIEF-5 scores of men with psychological ED were higher than those with organic causes, but there is no difference among patients with different organic pathophysiologies. Thus, IIEF-5 is not a definitive diagnostic tool to discriminate the pathophysiological causes of ED [135].

For men health PPPM-oriented questionnaires, the number of relevant data should be properly assessed and gathered into one patient’s profile as follows: data of sexual activity, libido, erectile function, difficulty achieving ejaculation, testosterone levels, and information regarding such factors as: BMI, chronic illness affecting hypothalamic–pituitary–testis axis, diabetes, CVD, neurodegenerative diseases, asthma, etc. in relation to lifestyle, sport, sauna, sport (bicycling), long-term strenuous work, mechanical trauma of testes, diet, alcohol consumption, smoking, environmental intoxication (medications, cadmium, etc.), exposure of radiofrequency fields, etc.

Relevant and proved biometric data, also self-measured, might be included to the questionnaires, e.g., for telemedicine consultations, if appropriately evaluated. Thus, among anthropometric and genital measurements, *anogenital distance* was proved to provide a novel metric to assess reproductive potential in men. A longer anogenital distance is associated with fatherhood and may predict normal male reproductive potential [136].

Construction of the intuitive *visual analog scale* (*VAS*)*-*based questionnaires are much preferable as such questionnaires have no significant differences with those, text-based, like IPSS [137].

### Couple health assessment within gender-related studies for family-oriented health programs

Considering biosafety of ceria nanoparticles, the group of potential patients (consumers) can be large and could be suggested to infertile persons of both genders [1]. Women health has been widely assessed within the large scope of factors affecting fertility, general health, with pathways among lifestyle, diet, obesity, and gender-related pathology [138, 139]. In men health, we still observe a lack of such focused attention in research and health care. Couple health assessment for smart planning social life and reproduction is a new challenge. Support of preventive educational activity with a long-term commitment of private and public funding programs is required.

### Endpoints

According to cumulated data and conclusions of several reviews (by Harris et al. [59], Drabovich et al. [69], Purvis et al. [84], etc.), we can assume as follows:

1. The male reproductive tract is exquisitely vulnerable to the effects of antiandrogens during development due to the reliance on the synthesis and action of androgens for the masculinization of the male reproductive tract [98].
2. An aging has a significant impact on male sexual function, sperm parameters, and fertility, which all contribute to decreased fecundability, increased time to conception, and increased miscarriage rates.
3. The trend for parenthood at an older age has also been seen in men [61, 62].
4. Older men tend to have older female partners, and increasing male age is associated with increased time to conception. This reflects the age-related increase in acquired medical conditions, decreases in semen quality, and increasing rates of DNA fragmentation seen in sperm.
5. The increased rate of DNA fragmentation leads to an increase in fetal abnormalities [59].
6. The level of testosterone does appear to influence sexual function. Declining testosterone may cause the decline in libido, erectile dysfunction, and difficulty achieving ejaculation.
7. Viral orchitis and sexually transmitted infections can lead to infertility due to germinal cell damage, ischemia, or the immune response to the infection.

#### Infection impact

8. The following are the conclusions regarding the role of infection in the etiology of male infertility from review by Purvis et al. [84]:

- Temporary inflammatory episodes in the male reproductive tract are probably common.
- A large number of men with poor sperm quality have been detected to have a nonsymptomatic, chronic prostatovesiculitis.
- Leukospermia or bacteriospermia are parameters for glandular infection.
- Increasing evidence implicates *Chlamydia trachomatis* as being a major cause of chronic nonbacterial prostatitis; an important aspect of *chlamydial* infections in men may be that the male accessory sex glands may function as reservoirs for the organism, increasing the probability of infection in the female.
- *Ureaplasma urealyticum* may also play an important etiological role in male infertility, but its significance is confounded by its acknowledged function as a commensal in the reproductive tract.
- One of the manifestations of male reproductive tract infection is the induction of sperm autoantibodies.
- There is a need for more systematic controlled studies of the effects of antibiotic treatment on sperm quality.

9. In/subfertility depend on many factors—internal and environmental. Moreover, environmental factors, such as pesticides, exogenous estrogens, and heavy metals may negatively impact spermatogenesis since male sperm counts were declined.
10. Cadmium intoxication is important in the etiology of varicocele-associated infertility.
11. Oxidative stress is most important in genesis in/subfertility; in addition, aging is also likely to further induce oxidative stress.
12. Metabolic syndrome, DM, and CVD have a significant impact on infertility.
13. Sports, hard work, smoking, soy-based food consumption, and radiofrequency fields could impact on a human reproductive health.
14. Multiparameter diagnostic assessment is essential (using broad panel with imaging molecular and clinical biomarkers).

#### Laboratory data

15. Key points for semen parameters were formulated by Drabovich et al. in their review in *Nature Reviews Urology* [69], as follows:

- “Seminal plasma contains a large number of tissue-specific proteins secreted by individual organs of the male reproductive system.
- Male reproductive system disorders result in altered composition of the seminal plasma proteome.
- High concentrations of organ-specific proteins enable their accurate quantification by mass spectrometry, facilitating biomarker discovery.
- As well as being a promising biological fluid for biomarker discovery, seminal plasma might find a unique niche as a clinical diagnostic fluid.
- Because cancer-specific proteins can appear earlier in seminal plasma than in blood serum, seminal plasma could facilitate the early diagnosis of prostate and testicular cancers” [60].
- Microdialysis might be useful to provide relevant tissue biomarkers in vivo.

#### Imaging data

- Ultrasound is a source of potential biomarkers, including:
- Scrotal ultrasound can provide relevant markers like testicle volume, blood flow (decline of perfusion, increased RI); testicular microlithiasis, tumors; seminal vesicles volume.
- Rectal ultrasound is useful for detecting prostate hyperplasia; prostatitis (calcifications fibrosis and cysts, compressing ejaculatory ducts), a nonsymptomatic chronic prostatovesiculitis, deep pelvic infections in the male; pelvic muscles dysfunction; and bladder hypermobility, etc.
- Transrectal ultrasonography-guided biopsy of reproduction organs and seminal vesicle fluid aspiration are important instruments to gather laboratory biomarkers.

#### Therapy approach

1. However, no effective *evidence level 1 supported* treatment has still not been seen on the horizon.
2. Low toxic and environmental safe nanodrugs with systemic multiorganic antioxidative properties are important to be implemented to the clinical practice.
3. Nanoceria being potential drugs that induce fertility have beneficial properties affecting collateral fertility modifying conditions due to their antioxidative, prokinetic, anti-inflammatory, antiviral, antibacterial, antifungal, and UV-protectant activity.
4. Properly randomized controlled trials are necessary to evaluate different subfertility treatments because pregnancies and spontaneous improvements in semen parameters occur without treatment that can find out whether pregnancy rates are higher or lower than expected after treatment [117].

### Study limitations

The main limitation of the study was small number of animals, however sufficient to obtain significant differences in most relevant parameter.

We have not assessed in the current research pregnancy rates, and the health of offspring as the most relevant outcome parameter of fertility, and have not included to the study the genetic factors, behavior, and sexual activity. Many bias and confounding factors have not been studied such as many health parameters including infections, BMI, etc. However, many factors missed in the methods were discussed through the paper. These issues should be further studied using properly developed infertility animal models designed in particular for young infertile rats related to collateral diseases affecting fertility with longitudinal assessment of molecular markers (using microdialysis) and imaging (using ultrasound, MRI, luciferase imaging) and also to test efficacy of nanoceria to enhance female reproduction. Drug delivery issues (theranostic, sonoporation) for infertility are the next essential points for a further research.

The issue of potential environmental contamination by nanoparticles due to their high penetration properties should be properly studied before extensive use of the nanoproducts.

### Consolidation of the PPPM Men Health concept

The results of the study are potentially applicable to create products, drugs and food supplements for treatment, to develop safe and effective treatments for age-related infertility beneficial for individual outcomes with particular application in elderly, to be a part of large concept within *interactome* in order to suggest sustainable healthy well-being and aging of men, and also to contribute in understanding many aspects related to male infertility and sexual life.

#### Preventive medical approach

Translation of the obtained data proceeding the animal model to a human organism may allow to consider to develop nanoceria-based additives/drugs against infertility in the particular application in elderly for treatment and preventive activity. The results have a potential for the prevention of spontaneous abortion with older paternal age and for the promotion of health in the integrated vision of *interactome*.

Screening, diagnosis, therapy, and prevention of male genital pathologies, such as varicocele, cryptorchidism, hernia, maldescent testes, testicular and prostate tumors, genitourinary infections, etc., study infertility and erectile dysfunction in the population involved in sports activities [121], identify adverse environmental and occupational risk factors, and correct underlying nutritional imbalances.

Another important fields recently discovered for PPPM approaches are essential to be applied in the matter, such as ecology medicine, development healthy individualized sports programs, diets, and support health-oriented fashion, dresses, furniture, transport design, and development sophisticated electronic gadgets [140].

#### Predictive medical approach

Smart reproduction planning and the planning of own person’s social life is the earliest and most important time point for effective predictive medicine for a future generation of a healthy society.

Men health in the point of view of reproduction and personal health and life quality is still not sufficiently studied and implemented to predictive assessment and widespread screening programs. Developing the panel of biomarkers for assessment age-related fertility for extensive vision of Men Health from the comprehensive view of including hypothalamic–pituitary–testis axis [141], psyche, stress, emotions, pain, physical activity, gut–brain axis (GBA), and molecular and cellular mechanisms is an important point. Development and validation of comprehensive and concise questionnaires for diagnosis male aging organism and sexual and reproductive life as a part of men health panel of biomarkers is strongly recommended.

#### Personalized medical approach

Designing person-related smart physiologic low-dose treatments is a challenge for medicine of the future. The clinical study is necessary for stratifications of potential responders to formulate clear personalized application. Considering biosafety of ceria nanoparticles, the group of potential patients (consumers) can be large and may be suggested to infertile persons of both genders.

Recent advances suggested that in order to create ex vivo systems for the generation of artificial sperm starting from immature germ cells from infertile patients, the personalized in vitro applications of nanoceria to enhance sperm quality and development of artificial sperm are allowed to be considered [142].

To suggest solutions to interact with industry to produce person-specific-shaped personalized fashion, dresses, furniture, transport design [140], and also via personalized manufacturing using 3D printing.

## Conclusions

In conclusion, the present work confirmed that administration for 10 days of citrate coated 2–5-nm CNPs lead to increase sex hormone levels, sperm count, and quality, as well as the activation of spermatogenesis in 24-month-old male rats. These changes were related to the improvement of serum oxidative–antioxidative balance.

### Expert recommendations

With the concluding points, we can formulate the following proposals (expert recommendations):

- To prepare an international research project to study the biomedical effects of nanoceria, capable to reduce the level of oxidative stress in diverse tissues of human body and potential as a drug inducing fertility with prokinetic, anti-inflammatory, antiviral, antibacterial, antifungal, and UV protectant activity.
- To initiate discussion to suggest the project with an extensive vision of Men Health within the comprehensive concept within PPPM for sustainable well-being and aging of men.

## Abbreviations

ANOVA: Analysis of variance
Ce: Cerium
CNPs: Cerium (Ce) oxide nanoparticles
DLS: Dynamic light scattering
LH: Leuteinizing hormone
LPO: Lipid peroxidation
ROS: Reactive oxygen species
SOD: Superoxide dismutase
TBA: Thiobarbituric acid
TEM: Transmission electron microscopy
XRD: X-ray diffraction
